# Arabidopsis Gene Family Profiler (aGFP) – user-oriented transcriptomic database with easy-to-use graphic interface

**DOI:** 10.1186/1471-2229-7-39

**Published:** 2007-07-23

**Authors:** Nikoleta Dupl'áková, David Reňák, Patrik Hovanec, Barbora Honysová, David Twell, David Honys

**Affiliations:** 1 Laboratory of Pollen Biology, Institute of Experimental Botany ASCR v.v.i., Rozvojová 263, 165 00 Prague 6, Czech Republic; 2 Department of Plant Physiology, Faculty of Science, Charles University in Prague, Viničná 5, 128 44, Prague 2, Czech Republic; 3 Department of Plant Physiology and Anatomy, Faculty of Biological Sciences, University of South Bohemia, Branišovská 31, 370 05 České Budějovice, Czech Republic; 4 Krymská 8/122, 101 00 Praha 10, Czech Republic; 5 Laboratory of Cell Biology, Institute of Experimental Botany ASCR v.v.i., Rozvojová 263, 165 00 Prague 6, Czech Republic; 6 Department of Biology, University of Leicester, Leicester, LE1 7RH, UK

## Abstract

**Background:**

Microarray technologies now belong to the standard functional genomics toolbox and have undergone massive development leading to increased genome coverage, accuracy and reliability. The number of experiments exploiting microarray technology has markedly increased in recent years. In parallel with the rapid accumulation of transcriptomic data, on-line analysis tools are being introduced to simplify their use. Global statistical data analysis methods contribute to the development of overall concepts about gene expression patterns and to query and compose working hypotheses. More recently, these applications are being supplemented with more specialized products offering visualization and specific data mining tools. We present a curated gene family-oriented gene expression database, Arabidopsis Gene Family Profiler (aGFP; ), which gives the user access to a large collection of normalised Affymetrix ATH1 microarray datasets. The database currently contains NASC Array and AtGenExpress transcriptomic datasets for various tissues at different developmental stages of wild type plants gathered from nearly 350 gene chips.

**Results:**

The Arabidopsis GFP database has been designed as an easy-to-use tool for users needing an easily accessible resource for expression data of single genes, pre-defined gene families or custom gene sets, with the further possibility of keyword search. Arabidopsis Gene Family Profiler presents a user-friendly web interface using both graphic and text output. Data are stored at the MySQL server and individual queries are created in PHP script. The most distinguishable features of Arabidopsis Gene Family Profiler database are: 1) the presentation of normalized datasets (Affymetrix MAS algorithm and calculation of model-based gene-expression values based on the Perfect Match-only model); 2) the choice between two different normalization algorithms (Affymetrix MAS4 or MAS5 algorithms); 3) an intuitive interface; 4) an interactive "virtual plant" visualizing the spatial and developmental expression profiles of both gene families and individual genes.

**Conclusion:**

Arabidopsis GFP gives users the possibility to analyze current Arabidopsis developmental transcriptomic data starting with simple global queries that can be expanded and further refined to visualize comparative and highly selective gene expression profiles.

## Background

Completion and annotation of the *Arabidopsis thaliana *genome represented a major step in plant genetic research [1]. This knowledge enabled gene prediction, assignment of functional categories and gave an opportunity to study gene and chromosome organization including the distribution of transposable elements. Finally it has enabled the characterization of global gene expression patterns at the transcriptome level at different developmental stages and under various physiological and stress conditions. Efforts to reveal the biological functions of thousand of genes and their integration into proteome, metabolome and interactome networks has become the principal focus of many studies and represents key objective of the 2010 Project [2]

A number of efficient and accurate gene expression analysis technologies to determine the expression levels of individual genes have been widely exploited in recent decades (Northern blot analysis, quantitative reverse transcription-PCR, cDNA library screening). Most of these methods enable analysis of the expression of single or relatively few selected genes. For the discovery of partial or whole gene functional or regulatory networks, the development of high-throughput technologies is essential with genome-wide transcriptomic studies providing a major input [3]. Several such methods have been developed including, cDNA fingerprinting [4], serial analysis of gene expression – SAGE [5], massively parallel signature sequencing – MPSS [6], high-density DNA oligonucleotide probe microarrays [7,8] or cDNA arrays [9]. DNA microarray technologies are among the most frequently used methods for parallel global analysis of gene expression. These methods are based on the principle of selective and differential hybridization between sample target molecules and immobilized DNA probes. Hybridisation to probes arrayed on a solid surface report the relative abundance of DNA or RNA target molecules by fluorescent signal detection [10,11]. Microarray technologies now belong to the standard functional genomics toolbox [12,13] and have undergone massive development leading to increased genome coverage, accuracy and reliability. Whole Genome microarrays developed by Affymetrix (Santa Clara, CA, USA) in collaboration with Syngenta represented the first standard in genome wide transcriptomic studies in plants. Whole genome Affymetrix ATH1 GeneChips cover about 76% of the *Arabidopsis thaliana *genes [14]. Moreover, the introduction of the Minimum Information About Microarray experiments (MIAME) as standard documentation for array experiments and in transcriptomic databases, increasing the value and comparability of microarray data [15].

The number of experiments exploiting microarray technology has markedly increased in recent years. Not surprisingly, there are potential difficulties in navigating between different available data sets. Microarray expression data are deposited on servers, many of which are publicly accessible. Public plant microarray data are deposited in several databases including ArrayExpress [16], GEO [17], NASCarrays [18] and the Stanford Microarray Database [19-21]. Currently these databases store several thousands of individual datasets and some of these offer on-line tools for data normalization, filtering, statistical testing and pattern discovery [22-26].

In parallel with the rapid accumulation of transcriptomic data, on-line analysis tools are being introduced to simplify their use. Global statistical data analysis methods contribute to the identification of overall gene expression patterns and to query and compose new working hypotheses based on these findings [11,12,27,28]. More recently, these applications are being supplemented with more specialized products offering visualization and specific data mining tools. Genevestigator, Botany Array Resource, Arabidopsis co-expression tool, and Expression Profiler offer Web-based tools to analyse large microarray datasets. Genevestigator offers two types of queries: a gene-centric approach and a genome-centric approach, which are represented by several analysis tools; Gene Correlator, Gene Atlas, Gene Chronologer, Response Viewer and the Meta-Analyzer, that is among the most sophisticated complex amongst available microarray analysis toolboxes [29,30]. Botany Array Resource offers similar services supplemented with tools for discovery and analyses of cis-elements in promoters [31]. Expression Profiler (EP) provides tools for hierarchical and K-groups clustering, clustering comparison, similarity search or the signature algorithm [32,33]. The more specialized PathoPlant database on plant-pathogen interactions and components of signal transduction pathways related to plant pathogenesis also harbors gene expression data from *Arabidopsis thaliana *microarray experiments to enable searching for specific genes regulated upon pathogen infection or elicitor treatment [34,35]. Finally, Arabidopsis Co-Expression Tool (ACT) allows users to identify genes with expression patterns correlated across selected experiments or the complete data set and offers the novel clique finder tool [36-38].

In this article we introduce Arabidopsis Gene Family Profiler (arabidopsisGFP, aGFP), a web-based gene expression database with visualization tools. During programming, we took into account that for many microarray data users, extraction of global expression patterns of single genes or gene families can be time-consuming and its visualization difficult. Moreover, the use of various normalization algorithms in individual experiments makes direct comparison of genes of interest within various datasets uncertain. To solve these issues, we developed aGFP to provide the user with two normalization and gene detection algorithms and a "virtual plant" Arabidopsis Gene Family Profiler facility that enables users to obtain a global expression profile of user specified and/or pre-defined gene families. These attributes of aGFP contribute a useful resource for the rapid bioinformatic analysis of Arabidopsis gene expression data through comparative expression profile analysis in a gene family-based context.

## Construction and content

The Arabidopsis Gene Family Profiler (aGFP) database was designed to give users the possibility to visualize expression patterns of individual genes, pre-defined gene families or user-defined gene sets in various tissues and at different developmental stages of wild type *Arabidopsis thaliana *plants. aGFP largely exploits microarray experiments obtained through the NASC AffyWatch transcriptomics service [39]. We adopted the general concept "from simple to complex". In the first approximation, an arithmetical mean expression signal from multiple experiments is displayed. In subsequent steps the user can choose to display expression data for individual plant organs or tissues at particular growth stages. This is accompanied by the option of progressive replacement of arithmetical means by individual expression values. So the user has the option to choose the different levels of visualization to suit needs. Finally, the user can switch from "virtual plant" visualization to a simple bar chart (standard or log-scaled) or tabulated display and can browse through individual experiments down to normalized or even raw data extracted from individual gene chips. Gene family data can also be visualized as a colorized spot chart.

Although the idea of web-based database tools is not novel, aGFP database offers a quick and interactive display of gene expression profiles using the virtual plant facility as well as alternate more conventional outputs. A novel feature of aGFP is that it enables the evaluation of the impact of normalization procedures on microarray expression data as well the possibility of rapid definition of user-defined families or gene groups. Simultaneously, aGFP serves as a facile and synoptic developmental reference guide for expression profiles of individual genes or gene families in wild-type *Arabidopsis thaliana *plants.

### Data resources

The arabidopsisGFP database covers transcriptomic experiments accumulated from wild type *Arabidopsis thaliana *plants of various ecotypes grown under normal physiological conditions. Original raw microarray data were obtained from Nottingham Arabidopsis Stock Centre (NASC) through the AffyWatch service [39]. In order to ensure the quality and compatibility of expression data only microarray experiments using Affymetrix ATH1 whole genome arrays with at least two biological replicates were included. To date, arabidopsisGFP database covers transcriptomic data from 345 microarrays covering 120 experiments.

### Programming

aGFP is composed as a relational MySQL database and Web server application, which is programmed in PHP script language [PHP:Hypertext Preprocessor]. Gene expression data are presented by dynamic HTML web pages with several types of graphic output. Graphs were generated using PHP module jpgraph [40]. HTML code was programmed to be correctly displayed in all commonly used internet browsers (Microsoft Internet Explorer/Mozilla Firefox/Opera). The user exploits a web-based interface for acquisition of custom-defined data. A user-friendly intuitive web-based interface is designed to enable simple and rapid navigation in aGFP. The aGFP database was created using general-to-specific strategy enabling the user to obtain a certain amount of information at every step with progressive targeted specification as the query develops.

### Data normalization

All gametophytic and sporophytic datasets were normalized using freely available dChip 1.3 software [41]. The reliability and reproducibility of datasets was ensured by the use of duplicate or triplicate hybridization data in each experiment, normalization of all arrays to the median probe intensity and the use of normalized CEL intensities of all arrays for the calculation of model-based gene-expression values based on the Perfect Match-only model [42,43]. A given gene was scored as 'expressed' when it gave a reliable expression signal in all replicates. An expression signal value of '0' means that the detection call value was 'absent' or 'marginal' in at least one replicate provided. In arabidopsisGFP, the facility is provided to instantly switch between transcriptomic data normalized by two different algorithms – MAS 5.0 or MAS 4.0.

### Annotation pages

Annotation of individual experiments is in accordance with MIAME standard [15]. *Arabidopsis thaliana *growth stages were according to Boyes et al. [44]. Affymetrix gene chips harbour several oligonucleotide probe types – prevalent unique probe sets (_at) accompanied by identical probe sets (_s set) and probes in a mixed probe set (_x set). Moreover, progressive Arabidopsis genome annotation has led to a reduction in the number of unique probe sets that has resulted in a reduction in the number of reliably 'present' genes. For these reasons, genes represented by these ambiguous probe sets were not included in the database [45]. This fact was taken into account and the aGFP database is regularly updated. Each locus in aGFP database is associated with relevant annotation released by TAIR (currently version 6) [46], and direct links to other web resources are available for each gene – TIGR [47,48], MPSS [49], TAIR [50,51], MIPS [52,53].

### Definition of gene families and superfamilies

arabidopsisGFP contains lists of pre-defined gene families and superfamilies enabling the rapid comparative visualization of expression profiles of their members. Genes in arabidopsisGFP are organized into two hierarchical levels consisting of gene families and superfamilies. All data were assembled from various relevant resources, the majority from TAIR – Arabidopsis Gene Family Information [54] and AGRIS [55,56]. Gene families were further organized in a different manner as gene family subclasses to different extent in each family and source. In order to simplify the different sub-divisions from different data sources, we rearranged them carefully and used only two levels, gene family and superfamily.

## Utility and discussion

### Data selection

At the aGFP home page, the user can select the search category (AGI number, BAC locus, Gene name, keyword) and two other input parameters; the gene detection algorithm (MAS4.0 or MAS5.0) and the source of expression data (AtGenExpress or NASCArrays). To make aGFP as comfortable as possible to use, at any stage of the query the user has the possibility to directly switch options between these pairs of parameters. This represents a distinct feature of the aGFP database that enables direct comparison of the influence of the detection algorithm or data resource on expression profiles.

aGFP database presents data normalised using two different algorithms, empirical MAS 4.0 and statistical MAS 5.0. Although MAS4.0 is believed to yields more false-positive calls [57], our analyses of four developmental stages of pollen development showed that the MAS5.0 detection algorithm tended to eliminate a number of genes originally detected as expressed by MAS4.0 and which were experimentally verified to be so [58]. This was often the case even for highly expressed genes (B. Honysová and D. Honys, unpublished results), highlighting the added value of the empirical MAS4.0 detection algorithm and comparative analysis.

**Figure 1:**
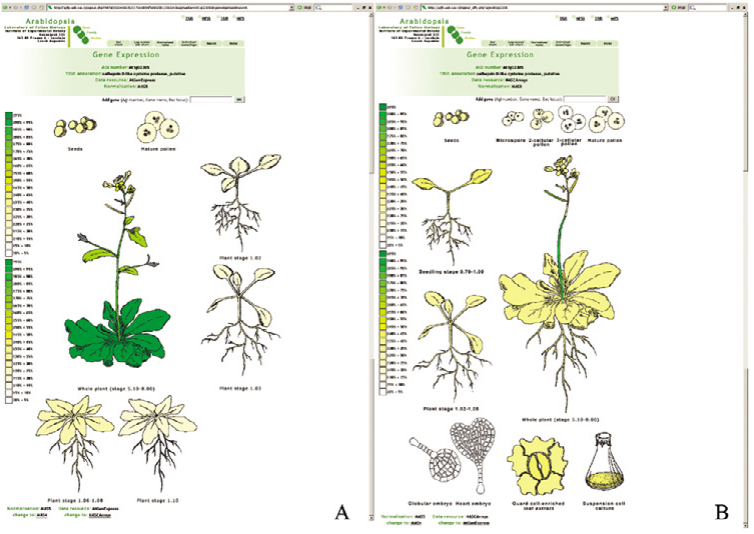
**Visualisation of gene search results using interactive "virtual plant"**. AtGenExpress (A) and NASCArrays (B) expression data for At1g02305 are shown.

Experiments included in the aGFP database are presented in two different subsets. The first subset contains data obtained within a scope of the AtGenExpress project [59], the second comprises all other datasets deposited at NASC and was labeled NASCArrays [26]. The reason for this separation was that AtGenExpress contains a structured set of experiments, involving Columbia-0 plants grown under comparable conditions to provide a gene expression atlas at several developmental stages. On the contrary, NASCArrays contains experiments carried out in various ecotypes grown under various conditions. Data in each subset are presented using several different graphical displays and, the user has an option to instantly switch between subsets in each environment (Fig. 1).

The other key feature of aGFP is the possibility to select pre-defined gene families and superfamilies. In subsequent steps, expression data for family members can be extracted down to the level of individual genes. Moreover, the user has also the possibility to work with custom-defined gene sets based on various search options (AGI number, Gene number, BAC locus, keyword search).

### Data visualization

**Figure 2:**
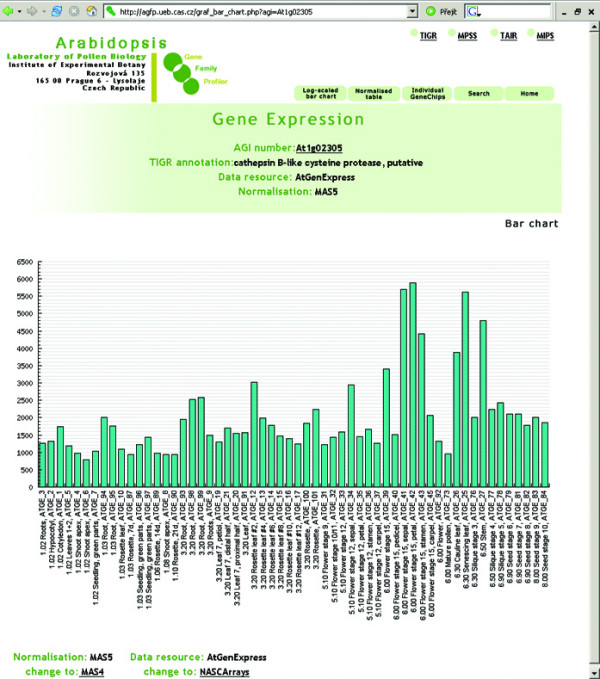
**Expression data visualisation**. Visualisation of At1g02305 gene expression using Bar chart option.

**Figure 3:**
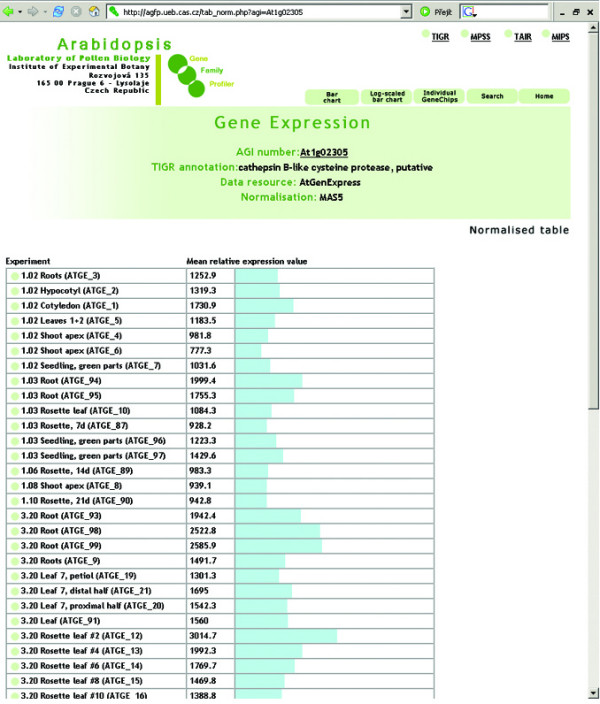
**Expression data visualisation**. Visualisation of At1g02305 gene expression using Normalised table option.

**Figure 4:**
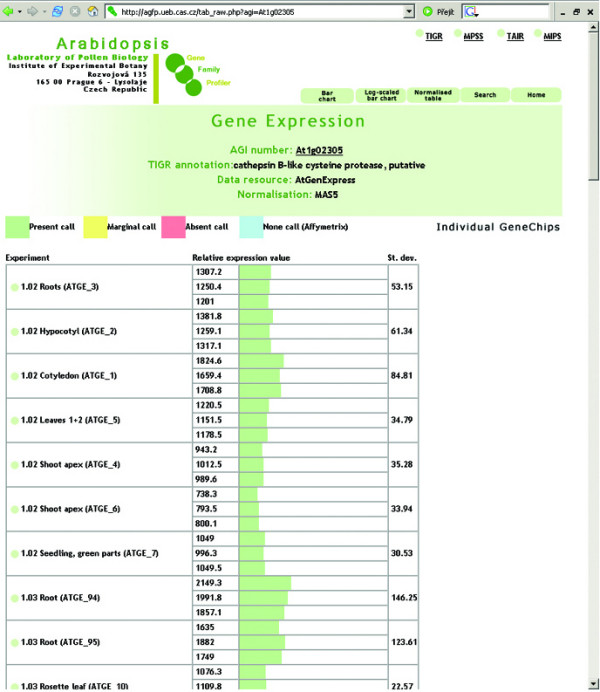
**Expression data visualisation**. Visualisation of At1g02305 gene expression using Individual GeneChips option.

**Figure 5:**
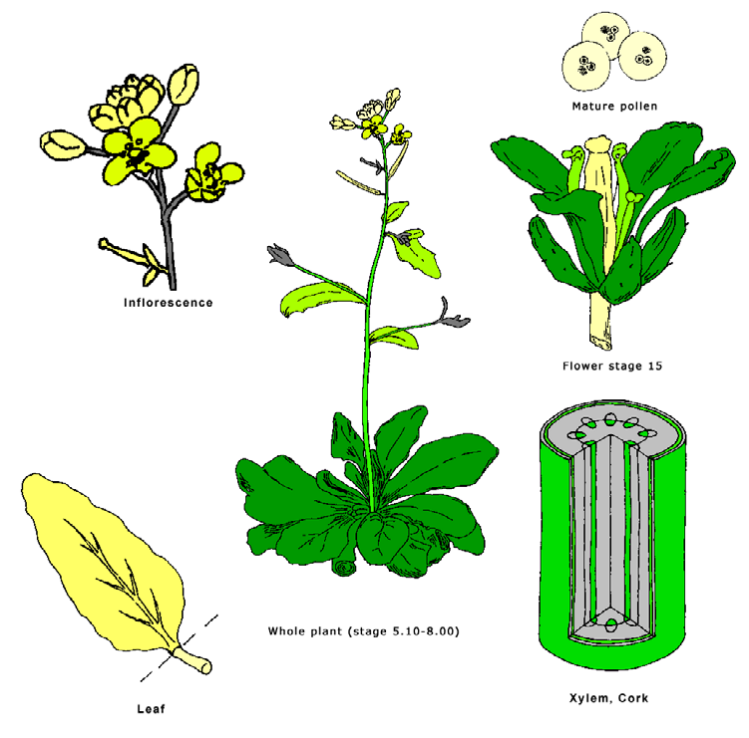
**Individual organs visualised by "virtual plant"**. Detailed expression profile of At1g02305 visualised in selected organs.

The aGFP database provides users with several different visualization formats. Apart from standard tables or bar charts (Fig. 2, 3, 4), an interactive virtual plant is used. The virtual plant comprises several growth stages defined according to Boyes et al. [44]. A white (low)-yellow-green (high) scale is used to depict the relative expression signals of individual genes, or gene families throughout the *Arabidopsis *life cycle. Mouse-over pointing to complex organs/tissues (i.e. flowers) causes opening of more detailed graphics showing individual organs (ie. sepals, petals, stamens, pistils and pollen; Fig. 5). This is in accordance with the adopted aGFP database concept "from simple to complex".

**Figure 6:**
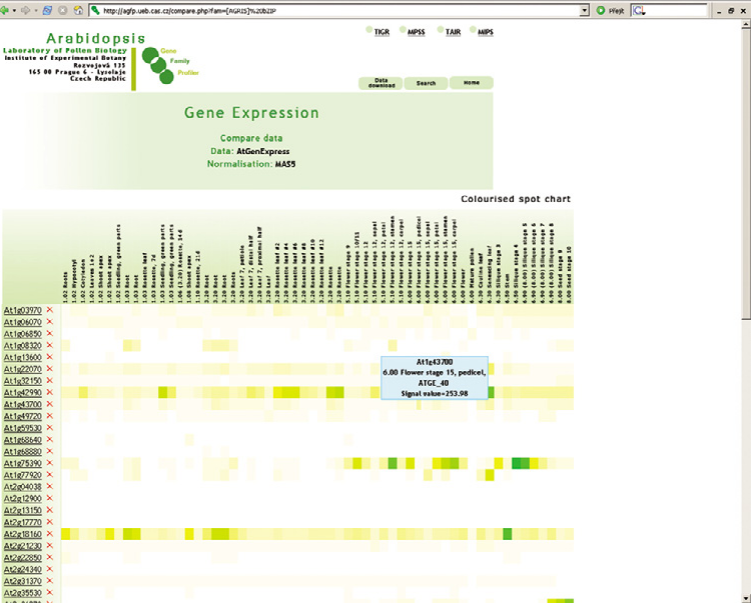
**Colourised spot chart visualization**. Visualization of expression profiles of bZIP gene family members (AtGenExpress).

In addition to the virtual plant and bar chart graphics, expression profiles can also be visualized as interactive colorized spot charts or heat maps. The colorized spot chart uses a colour scale identical with that of the virtual plant (Fig. 6). The interactivity of modeling is based on the possibility of ad hoc addition and removal of genes to and from a currently active set. Moreover, each spot contains information about expression signal value and experiment that is activated by mouse-over. Therefore visualized expression data are not merely summarized, but instead represent direct output from individual microarray experiments.

### Data accessibility and legend

The complete datasets used in the aGFP database are described in the Legend available from the homepage and it is possible to trace the origin of all datasets. Moreover, data can be downloaded for individual and selected gene sets as a TAB-delimited text. This enables the direct import of downloaded data into spreadsheet editors such as Excel and database software such as Access and FileMaker. This text file contains a list of developmental and morphological stages, normalized expression data for the selected normalization algorithm and data source.

## Conclusion

arabidopsisGFP is a microarray expression database of wild type *Arabidopsis thaliana *plants grown under physiological conditions. It gathers data from experiments using Affymetrix ATH1 whole genome arrays with two or more biological replicates. From the outset, it has been created as intuitive user-oriented web-tool employing a "general-to-specific" concept enabling the user to obtain certain amount of information at every step with progressive specification and refinement. The aGFP database contains several gene selection and grouping tools including pre-defined gene families. It also provides the user with different gene expression visualization options including a unique "virtual plant" graphic display. Easy switching of visualization options gives the user the possibility to rapidly select the most suitable form of data presentation. A novel advantage of the aGFP database is the provision of alternative normalization treatments of microarray data using statistical (MAS5.0) and empirical algorithms (MAS4.0). Together with the facile switch between these detection algorithms it provides the opportunity to instantly assess the reliability of gene expression data. Arabidopsis Gene Family Profiler represents a versatile tool for facile visualization of transcriptomic data that can be exploited in genome-led queries of gene and gene family functions and regulation.

## Availability and requirements

The aGFP database is freely accessible and its concept offers the possibility to extract and visualize expression profiles of individual genes, gene sets, gene families or gene superfamilies from a broad spectrum of microarray experiments covering various *Arabidopsis *organs, tissues and developmental stages. For these purposes, an innovative graphic concept of the "virtual plant" was introduced representing a clear and simple visualization of gene expression profiles in a morphological and developmental context. The arabidopsisGFP database is accessible at .

## Authors' contributions

ND, DT and DH defined the concept of arabidopsisGFP database. ND and PH programmed all scripts. DH is responsible for data normalisation. DR worked on gene families and superfamilies definition. DH, DT, ND and BH specified the data visualization concepts and BH drew the "virtual plant". All authors read and approved the final manuscript.
